# Barriers and facilitators in the implementation of mobilization robots in hospitals from the perspective of clinical experts and developers

**DOI:** 10.1186/s12912-023-01202-2

**Published:** 2023-02-17

**Authors:** Angelika Warmbein, Ivanka Rathgeber, Janesca Seif, Amrei C. Mehler-Klamt, Lena Schmidbauer, Christina Scharf, Lucas Hübner, Ines Schroeder, Johanna Biebl, Marcus Gutmann, Inge Eberl, Michael Zoller, Uli Fischer

**Affiliations:** 1 Clinical Nursing Research and Quality Management Unit, University Hospital LMU Munich, Marchioninistr. 15, 81377 Munich, Germany; 2 Professorship of Nursing Science, Faculty of Social Work, Catholic University of Eichstätt-Ingolstadt, Eichstätt, Germany; 3 Department of Anesthesiology, University Hospital LMU Munich, Marchioninistr. 15, 81377 Munich, Germany; 4 Department of Orthopedics and Trauma Surgery, Musculoskeletal University Center Munich (MUM), University Hospital LMU Munich, Munich, Germany

**Keywords:** Robotics, Implementation, Technology, Inpatient setting, Nursing, Integration, Innovation, Hospital

## Abstract

**Background:**

Early mobilization can help reduce severe side effects such as muscle atrophy that occur during hospitalization. However, due to time and staff shortages in intensive and critical care as well as safety risks for patients, it is often difficult to adhere to the recommended therapy time of twenty minutes twice a day. New robotic technologies might be one approach to achieve early mobilization effectively for patients and also relieve users from physical effort. Nevertheless, currently there is a lack of knowledge regarding the factors that are important for integrating of these technologies into complex treatment settings like intensive care units or rehabilitation units.

**Methods:**

European experts from science, technical development and end-users of robotic systems (n = 13) were interviewed using a semi-structured interview guideline to identify barriers and facilitating factors for the integration of robotic systems into daily clinical practice. They were asked about structural, personnel and environmental factors that had an impact on integration and how they had solved challenges. A latent content analysis was performed regarding the COREQ criteria.

**Results:**

We found relevant factors regarding the development, introduction, and routine of the robotic system. In this context, costs, process adjustments, a lack of exemptions, and a lack of support from the manufacturers/developers were identified as challenges. Easy handling, joint decision making between the end-users and the decision makers in the hospital, an accurate process design and the joint development of the robotic system of end-users and technical experts were found to be facilitating factors.

**Conclusion:**

The integration and preparation for the integration of robotic assistance systems into the inpatient setting is a complex intervention that involves many parties. This study provides evidence for hospitals or manufacturers to simplify the planning of integrations for permanent use.

**Trial registration:**

DRKS-ID: DRKS00023848; registered 10/12/2020.

## Background

Severe illnesses such as strokes or cancer have increased immensely worldwide in recent decades [1, 2]. These diseases require intensive treatment, which is usually carried out in an inpatient setting in the initial stages. In addition to specialist medical care and medication, specialized therapeutic interventions can positively influence the healing process, for example, nature-based treatments [3] or exercise-related interventions.

Some studies have already shown that early mobilization may have a positive effect on the healing process [4–6]. The definition of early mobilization varies [7]. In acute care hospitals, where most initial treatment is provided, the focus of therapy is on surgery and/or medication. In addition, according to the German S2e guideline “Positioning therapy and early mobilization for prophylaxis or therapy of pulmonary dysfunctions” [8], patients receive exercise therapy, which is carried out by nursing services and physiotherapists.

In practice, many intensive care patients receiving intensive care suffer from intensive care unit (ICU)-acquired weakness or muscle atrophy [9, 10]. Studies have shown that intensive mobilization training could provide an opportunity to reduce some of these side effects [11–13] and might reduce the length of hospital/ICU stay [14]. However, a high frequency of mobilization is difficult to achieve in practice due to a lack of staff and time [15, 16], and the high physical effort required in mobilizing sedated patients causes the frequency and intensity of mobilizations to decrease [15]. Additionally, health and safety risks [17], for example, transferring the patient to a separate exercise device, are major barriers to performing mobilizations.

One way to reduce the physical strain on mobilizing professionals and to increase the frequency and intensity of training sessions is to use modern technologies such as assistive robotics. In the context of research, robotic-assisted mobilization has already been evaluated for its added value [18–21], but the focus is almost exclusively on patient outcomes [22, 23]. The difficulty of integrating new technologies and thus new processes into a highly complex environment such as intensive care has only been examined within certain hospitals [24]. However, understanding the interplay between innovation and the local environment [25, 26] is crucial for successful implementations in practice, as complex innovations in nursing lead to changes in existing processes [27]. Therefore, it is essential to identify influencing factors and include them in development and implementations [28]. So far, an overview of the experiences of integrating robot-assisted mobilization therapy into inpatient settings is still lacking.

### Aim

This study aimed to provide an overview of the barriers and facilitators to the implemention of robotic systems for mobilization therapy into inpatient settings like intensive care or early rehabilitation facilities. We describe which circumstances as well as environmental and person-related factors need to be considered to facilitate implementation.

## Methods

### Design and setting of the study

This preliminary study was part of a three-year research project conducted under the Medical Research Council’s [29] framework for development of complex interventions and took place in the phase of development. A qualitative approach was chosen following the Consolidated Criteria for Reporting Qualitative Research (COREQ) [30] (see attachment). An exploratory qualitative design with a single data collection point was chosen using a semi-standardized, topic-centered interview guideline. Interviews were conducted with European experts from the fields of practice, science and development to gain a deeper insight into the integration of robotics for assisted mobilization. The experts’ robotic systems were designed, evaluated, or manufactured for inpatient care with a focus on physical rehabilitation.

### The research team

The research team consisted of senior researchers, PhD students, and other scientific colleagues. Most of the researchers directly involved in the data collection and analysis have worked as trained nurses. The interviews were conducted by trained female researchers and were pre-tested. No relationship had been established with the respondents prior to the study.

### Characteristics and recruitment of participants

In order to obtain information regarding the barriers and facilitators in the implementation of robotic systems, we first contacted purposively identified professionals and then added further professionals using the snowball method. A total of 26 individuals were approached by phone and email via research networks and internet research. Explicit inclusion and exclusion criteria were defined. To include experts in the study, the individuals had to (a) be conducting research on, (b) be developing motion-related robotic systems, or (c) be involved in at least one integration of robotic systems for health-promoting, inpatient settings (like rehabilitation units or hospitals). This could be, for example, inpatient patient care; the development, production, and distribution of robotic systems; or the research of robotics for physical health promotion. Through the multi-professionalism of the experts, a one-sided view should be avoided and the questions be addressed more precisely. The experts had to be proficient in German or English and have been working in their field for at least three years. This inclusion criterion is intended to ensure that the experts are firmly established in their work environment and that any barriers and support factors seen are exclusively attributable to their handling of robotics. Experts who had experience in other areas of robotics or who had been active in their field for less than three years were excluded. A total of 15 individuals agreed to participate. Eleven individuals did not respond to the interview request. The potential participants were sent the information leaflet, the consent form, and a factsheet about the overall project of which the study was a part. The documents provided information about the purpose, personal rights, and data protection. Additionally, the themes of the interview guide were shared with the participants. Of the 15 potential participants, one interview with two individuals had to be discontinued because they did not meet the inclusion criteria. All participants gave informed written consent.

In total, 11 interviews with 13 experts were conducted. Two interviews were carried out with two interviewees at a time.

### Data collection

The first and the second authors (AW and IR) conducted the interviews. Both authors work in projects concerning the implementation of robotics into nursing services. The first author has previous experience in conducting qualitative research, and the second author has a nursing background and was trained in interviewing. Both authors pre-tested the interview guidelines for practicability, aims, and wording and discussed these within the research team. Due to pandemic conditions, all the interviews had to be conducted via online video tools. Data were collected from December 2020 to February 2021.

The interview guide provided the thematic structure: First, the interviewer provided structured information about herself and about the study/project. After an additional short clarification regarding the interviewee’s rights and data protection, a socio-demographic questionnaire was filled in together by the interviewee and interviewer. In the questionnaire, questions regarding gender, age, setting (inpatient care, science, and development/manufacturing), job title and qualification, duration of activity in the sector, and duration of the handling of robotic systems were asked. The answers were used to map the characteristics of the study population.

Afterwards, the interview was conducted using a semi-standardized interview guide [31] with the following topics:

- Previous experiences with integrations of robotic systems,
- Experienced supporting factors in the integration (structural, person-related and environmental factors).
- Experienced barriers in the integration (structural, person-related and environmental factors).
- Used solutions to overcome barriers.

The questions were designed to be open-ended. Topics mentioned by the experts were explored in greater depth. After the interview, field notes about the atmosphere and interruptions were written down. Interviews were conducted until the statements of the interview partners became repetitive and no new insights could be gained (data saturation) [32].

### Analysis

The interviews were recorded using a recording device and transcribed verbatim using MAXQDA 2022 Software [33]. Analysis was carried out using latent content analysis [34]. Within the coding process, open coding, axial coding, and selective coding were performed [35]:

- Audio records were transcribed verbatim and cross-checked by the research team.
- In the first step, open coding was performed. These codes were evolved in a two-stage process according to the method of meaning condensation [36]: Three researchers coded the transcripts separately and discussed the developed codes until agreement was reached. Where discrepancies occurred, a senior researcher was involved.
- Afterwards, the codes were grouped (axial coding) according to themes. and discussed within the research team.
- These coding groups were summarized into several main themes.

### Rigour

The study was conducted according to the quality criteria of openness, flexibility and processuality, intersubjectivity, comprehensibility, appropriateness to the subject matter, and limitations [37, 38]. Within this study, credibility, transferability, confirmability and dependability were assured [39]. Credibility was achieved by conducting at least two pre-tests of the interview guide per researcher as well as comprehensive preliminary research on the topic and the field. Participants were comprehensively informed in advance about the objective and the topic areas. To improve transferability, the results of each step of the analysis were discussed by at least two researchers until agreement emerged [40]. Dependability was achieved by detailing the steps of data collection, analysis, and research design in the study. Confirmability was ensured by following the participants’ thematic focus in-depth.

## Results

### Study population

The experts came from the industry and development of robotic systems (31%), from research (23%), and from practice or clinical settings (46%). Five were female, and eight were male. Seven were in a management position. Eight individuals were from Germany and five were from Denmark (1), Austria (1), or Switzerland (3). The interview participants were on average 45 years old and had been working in their respective industry for an average of 17.5 years. On average, they had been in contact with robotics for 8.8 years. Nine interviewees had an academic degree in healthcare or engineering, and four had completed a professional training as a nurse or physiotherapist. The atmosphere of the interviews was mostly friendly and neutral; in two interviews, the atmosphere was reserved but friendly. Some interviews had been interrupted by colleagues of the interview partners. The interviews had an average length of approximately 36 min. The experts talked about integrations of robotic systems used in physical rehabilitation. The various devices either trained individual muscle areas (such as legs or arms) or were geared toward whole-body training. The majority was used in acute therapy, such as stroke treatment or postoperative early mobilization training. The robotic systems were either loaned for testing, leased for a certain period of time or sold to hospitals.

**Fig. 1:**
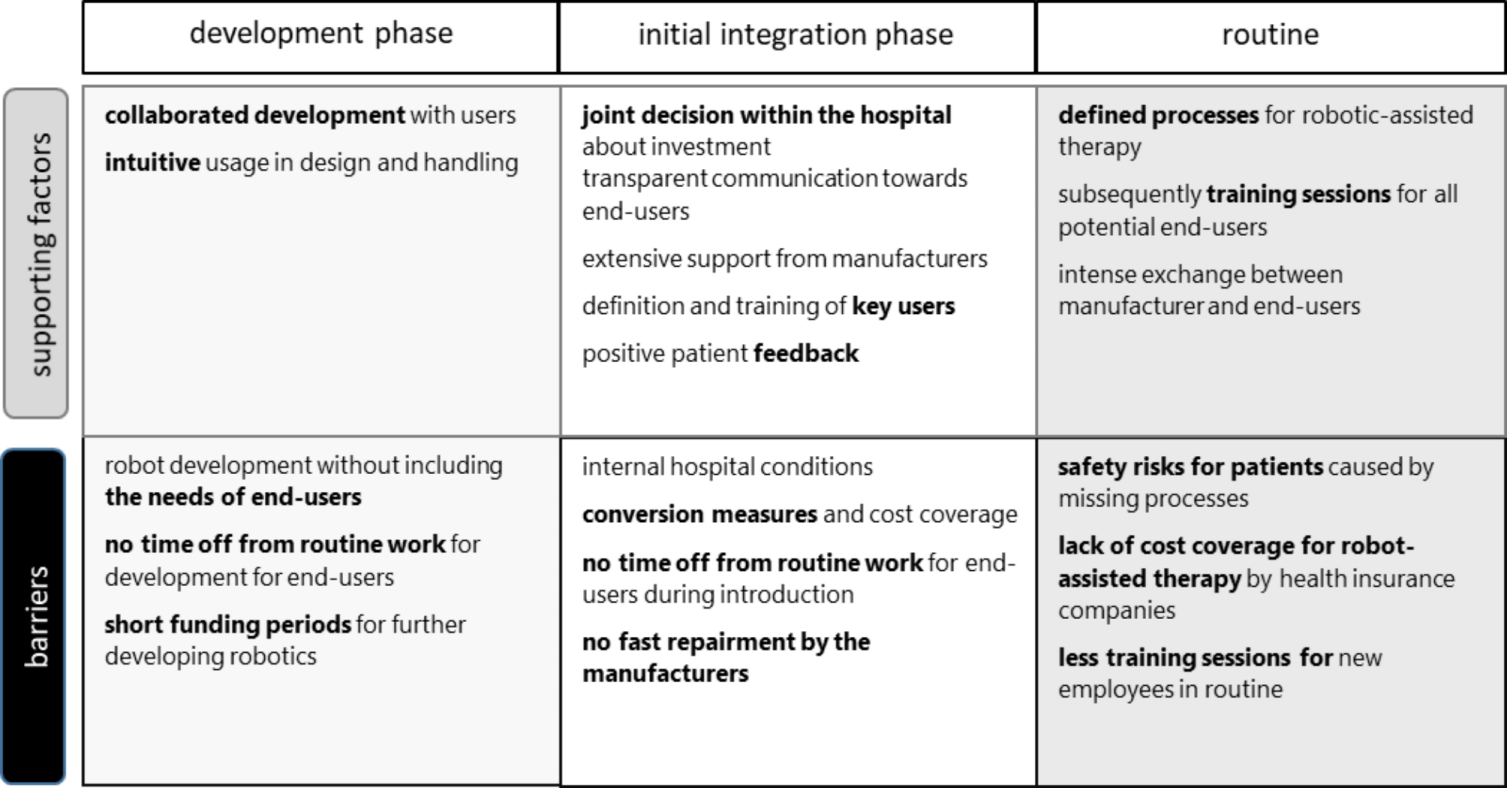
Clustered overview of results according to stage of integration and support factors and barriers

The results were assigned to three stages of integration into the acute clinical setting: development (before integration), in the initial introduction, and routine usage. For these phases, the experts explained which points were beneficial for longer-term usage on patients and which factors had a negative influence to the point of failure of the integration (see Fig. 1).

### Development phase

In the implementation of a robotic system, many experts took a step back and described the important factors influencing integration that had already taken place in the development phase of a robotic system. In this phase, functionality and collaboration were important factors influencing future usage.

#### Supporting factors

Collaboration between end-users and manufacturers was described as beneficial for adaptations to clinical requirements and small additions. These additions were not necessarily related to the therapy, but could have been based on a simplification of the handling procedures (e.g. adding trash cans to the robotic system in order to avoid extra walking distances).

*“[The end-users] had a whole wish list that we [the technicians] basically worked out with them at the very beginning. […] We created the development process and the robot in such a way that we come closest to their requirements.“ (I3 scientist)*.

In parallel to the collaboration in initial development, the constant exchanges during events and trade fairs in the form of feedback loops were also considered to be very positive.

*“Basically, we [the technicians] have also received feedback directly from the market on this and overall very high feedback on the way it is implemented.” (I3 scientist)*.

#### Barriers

Robotic systems developed separately from the clinical setting were not practical enough to be used permanently. This may be due to their handleability in general or the non-adaptability to the different conditions of the patient population.

*“It is always nice if the scientists and engineers come up with a solution, but then somehow often develop it missing the point of the market. They often forget to take the opinion leaders [of the end-users] on board.“ (I1 manufacturer*).

The non-participation of nurses in the development was often caused by a lack of time off in their daily work. A further aggravating factor was the lack of education regarding technology and robotics within the training to become a nurse or physiotherapist, practioners and manufacturers stated.

Another hurdle for the manufacturers and scientists was too little public funding and short funding periods for the development of a robot adapted to practical needs, as well as difficulties in obtaining approval for it.

### Initial integration phase

The experts elaborated in detail about the manufacturer’s support, internal hospital processes and adaptations, and financial issues within the integration phase.

#### Supporting factors

Before integration into the clinical environment, the hospital and the manufacturer had to make certain preparations. Moreover, the decision for robotic therapy devices had to be supported by financial managers and the clinic’s management, team leaders, and employees. The decision makers’ incentive focused more on the economic factors of the personnel for offering more therapies and using them as an “*exposed promotional tool” (I11 practioner).* For the end-users, a visible effect on patient recovery and a relieved burden in performing therapy were important. Motivated and open-minded end-users also enthused employees with less technological knowledge via the snowball effect.

*“That worked out quite well, so it’s starting to spread, and people are starting to influence each other on these things.“ (I2 manufacturer)*.

Within the hospital and the departments, open and transparent communication was helpful. It was clearly communicated to users that robotics are tools or aids that would not put jobs at risk. This form of communication was also used across departments in some hospitals to create an open atmosphere toward new technologies.

“*The robot could also be considered as a Swiss pocket knife. So, as an aid for solving the tasks properly, for support and not to replace. A robot should not and cannot do that*.“ (I5 manufacturer).

Intensive support from the manufacturer with extensive training and follow-up appointments within the following two weeks was important. At the beginning of the rollouts, team leaders and employees were provided with informational materials and motivated employees were invited to become key users. These employees became the main users within the departments and were part of the “*in-house expert panel*” *(I11 practioner)* or “*core team*” *(I10 practioner).* It was considered positive if the hospital gave the employees time off for the integration or testing phase and when there was guidance from more experienced users or the manufacturer as a safety mechanism.

It was crucial for employees to be able to test the device on themselves. Through their own experience, employees were able to assess how to design the training for patients.

*“It really helped that we were all allowed to train with the robot ourselves, so the combination of watching but also experiencing walking in it was very positive. So you can really feel the difference to normal walking, or, rather said, how close it comes to normal walking.“ (I12 practioner)*.

Clear expectation management regarding usability and limitations was also helpful. By setting realistic goals, the idea that “*false expectations are stoked*“*(I12 practioner)* was prevented.

Moreover, robotic-assisted therapy emerged as a form of physical relief for the users. Robotics generated a higher frequency of movement sequences, which, without robotics, would only have been possible with greater physical effort.

*“[Robotics] can make everyday life easier for me. […] In early mobilization, I would have had to work with greater physical effort for a leg movement. So it has to be brought to the foreground that [robotics] is also a physical relief for* us.“ *(I4 practioner).*

Additionally, nurses preferred to use robotics if the patient feedback was positive.

#### Barriers

Challenges to integration included internal hospital processes and structural conditions, costs, technical defects of the robot, patients, and employees.

Internal hospital processes such as hospital hygiene, work safety, and data protection requirements, as well as technical conditions (stable WLAN coverage and power supply), made integration difficult. The premises were also not designed for the integration of robotic systems: room sizes, noise levels, floor load, and heat generation were problematic, so additional costs had to be invested for conversion measures.

“*The robot was first installed in a room for medical training therapy until they realized that it was actually too heavy for the floor and that they had to put extra plates under it.“ (I12 practioner)*.

In cases where the devices were used at the patient’s bedside or in intensive care units, conversion measures were not possible. This also ruled out larger robotic systems from the very beginning, although there would have been interest in the device.

Cost coverage was also challenging for the hospitals. The robotic devices were usually purchased or rented by the respective hospital alone without subsidies or contributions from health insurance companies.

*“The robot, as well as the rent or the purchase, costs a considerable sum; you will never be able to cover the costs.“ (I10 practioner)*.

Additionally, structural hurdles arose in the integration process in the hospitals. Integration into daily workflows proved difficult, with robot-assisted therapy taking up more time than conventional therapy or disrupting daily routines due to noise or lack of space. No extra time off for key users also led to one integration failure.

*“The project just falls apart. There is nothing you can do. If there is no structurally adequate integration over a longer period of time, then it won’t work.“ (I1 manufacturer)*.

The human factor also played a major role. In the user teams, it was a hindrance if the team leadership was not supportive of the integration. Likewise, it was noted that some professionals permanently rejected the use of robotics because they “*only want to work with their hands, don’t want anything modern on it” (I12 practioner).* Often, there was also a lack of safe handling or a fear of being replaced by technology.

*“There were also many fears ‘Okay, now technology is coming into the house, it will make my job redundant’.“ (I11 practioner)*.

Integration was also hampered when professionals could not see a positive effect on the patient’s healing process or a workload reduction.

Furthermore, some patients did not want to work with robotics. These patients required intensive care, were elderly, or had concomitant psychological factors. Skin damages like skin tears initially appeared due to the use of one robotic device.

Additionally, outages, non-timely repairs, or the lack of extensive support from the manufacturers presented a hurdle for integration. This was mostly put in relation to the high investment costs. Similarly, it was noted that outages led to negative feedback from patients.

*“When some part of the robot breaks and you call [the technicians], […] someone has to be there the next day to fix it. It should not take a longer period of time.“ (I12 practioner)*.

### Routine

Some experts named factors that affected the use of the robots after the intensive introductory phase. Station processes, training, and costs were highlighted.

#### Supporting factors

Standardized processes such as a fixed group of users or a rotation for robot-assisted therapy were claimed to be useful. It was seen as beneficial if specialist supervisors were involved.

*“A stable robotics operation is normally given, if there are responsible persons. These persons focus on robot-assisted therapy and spend most of the day with it.“ (I12 practioner)*.

Key users eased the start for the other nurses, who all subsequently also received training. Usually, one employee supervised a single robot-assisted mobilization, even when multiple devices were available. In one hospital, employees were so familiar with the devices that they were able to perform four robot-assisted therapies simultaneously. So, more therapies could be carried out in the same amount of time. Since therapy units could be billed (to insurance companies or private persons), an additional source of revenue was generated. The simultaneous operation and therapies were rarely reported by the other experts.

Adapted support, periodic visits, and integrating users’ feedback into further development by the manufacturers was seen beneficial. They also created the opportunity for exchange between hospitals through annual workshops so that ideas could be jointly formulated and further thought through by the users.

#### Barriers

Challenges arose if the robotics were not integrated into processes of the respective ward from the beginning- especially in acute or intensive care wards, where highly complex interprofessional care was provided. Standardized processes could refer to responsible professionals or defined therapy times. If these standardizations were missing, subsequent integration was made more difficult. Safety risks due to a lack of defined processes or short application times were the possible results.

*“If I say right from the start, well, you can have this leeway, then experience has already shown me that at some point ‘ah yes, it’s not so bad’. And then the three degrees difference, quickly change to that ‘I can allow a few more’.“ (I4 practioner)*.

In the case of mechanical occurrences of the robot, conventional therapy was mostly preferred because it is more controllable and closer contact with the patient is possible.

“*And once that starts to get bogged down because you’re potentially deciding ‘do I take this therapy route or that one?‘ So, then therapists often decide against the devices.“ (I12 practioner)*.

Hospitals were facing the challenge of implementing robotics in terms of cost coverage. A problem arose in the accounting with health insurance companies, as the robotic-assisted therapy had not yet been firmly integrated into the cost coverage catalog.

In the ongoing process, the training of new employees also presented a hurdle. In their work routines, no elaborate training programs or time off were provided for individual employees.

*“If the device has been in the house for some time, then the familiarization period is usually significantly shortened compared to a new introduction. […] Then I guess you have to be very, very careful.“ (I4 practioner)*.

This may lead to users being underprepared for intensive therapies for vulnerable patient populations, such as those in early rehabilitation.

## Discussion

This study points out that the implementation of robotic assistance systems in acute inpatient care settings requires profound preparation and structuring due to a variety of underlying reasons.

Nursing care in acute inpatient settings, especially intensive care, is highly complex and characterized by situational flexibility regarding the patient’s condition. Workflows are built on standardized processes that follow national guidelines [8] or have been developed internally within wards/hospitals. A clear process description is also essential for robot-assisted mobilization, according to the experts in this study. These should be accompanied by implementation science frameworks such as Consolidated Framework for Implementation Research (CFIR) or Theoretical Domains Framework (TDF) [41]. Without this, integration into daily routines is difficult, as Bertelsen also described [24] and challenges may arise in terms of end-user acceptance due to missing knowledge, attitudes and resources [27].

Defining clear responsibilities, such as forming a core team, also creates structure in the area of staff planning. Nurses in the core team should be given time off for integration, according to the experts’ recommendations. This could be achieved, for example, in a manner similar to wound management in Germany. These nurses are specialized and work either across hospitals exclusively in the area of wound care or as specialists within a ward [42, 43]. it would imply an adaptation of the nursing staff structures to innovative technologies. However, specialization in robot-assisted therapy and separating these specialists from the ward team would require new thought processes and further research. Thus, there is a possibility that problematic aspects such as less mobilization due to a lack of time or acute staff shortages [15, 16] may occur to a lesser extent. Additionally, if task assignment is regulated within the ward team in the form of the core team, risks such as the lack of safe handling of robotic systems can already be reduced.

Studies have demonstrated that mobilization is performed less when it involves more physical effort [15]. Various robotic systems offer the possibility of minimizing physical effort by taking over the lifting and supporting tasks. This was also pointed out by experts from the field, who linked the statement to more intensive training for the patient. The positive effect of robot-assisted mobilization on patient well-being also affected the motivation of the users according to the experts. Just et al. [22] were able to show positive trends in patient outcomes, but further research is needed to generalize this statement.

The development of robotic assistance systems has made considerable progress in recent years. However, there is a discrepancy between the requirements of clinical practice and the technical developments to implement and use robotics intuitively and without increased additional effort. Similarly, the clinical requirements for a medical device to be used on patients are a significant hurdle. The experts emphasized that exchanging opinions with users during the development phase is essential for this. Vermeesch et al. [44] pointed out that, in connection with robotics, certain conditions must be satisfied to allow the patients and users to rate the device as acceptable and useful. Kerssens et al. [45] found that caregivers have high expectations of robotics in home care. In the expert interviews, this was similarly confirmed for the acute inpatient sector, showing that a human-related barrier arises if the demands are not met.

Human-related barriers were also mentioned by the experts with regard to other aspects. These included, in particular, team dynamics, affinity for technology, and the motivation of the individuals. Servaty et al. [46] also described a lack of motivation as one of the biggest hurdles for implementation. Waibel et al. [47], however, could not identify any significant hurdles in this respect of their qualitative study.

Similar to the expert survey, this study also highlighted the costs of purchase and permanent financing [47]. The experts increasingly emphasized that financing of the devices for permanent implementation represents is an economic challenge. In addition to the purely monetary expenditure for the device, this also includes any measures that need to be taken structurally. Grunow et al. [48] suggested that sufficient space for robotic devices should be included in the design of patient rooms in the intensive care setting.

The rapid development in the field of robotic systems requires further research on various aspects to further optimize patient care and to provide nurses with future-oriented devices, especially the influence of various human, technical and structural factors on the success of an implementation [26].

### Limitations

Due to the higher number of experts from Germany, many results refer to inpatient settings in this country and may therefore not be transferable to other settings or countries. Also, the small and heterogeneous sample might give a limited insight into the topic. Since this study dealt with integration processes into the clinical setting, only professionals had been included. The patients’ point of view was disregarded, as they could only reflect unique therapy experiences and it was assumed that caregivers incorporated their patients’ feedback into therapy and into their statements. Statements by a great cohort of patients would have strengthened the research and should be integrated in following research.

### Implications

Many factors must be taken into account before purchasing/renting robotic devices for use in nursing practice [49]. Hospital and ward managers should consider in advance whether the conditions (such as space, floor conditions, and noise levels) on site are suitable for the robotic device and whether the costs can be covered without subsidies from health insurance companies. Technicians have to ensure that the robot meets the requirements for clinical use as part of the development. Within the integration process, it is essential that a core team of nurses is trained. These key users should be motivated and support the implementation. Moreover, standardized processes need to be developed for robot-assisted therapy. These can be ward-related or hospital-related and should be generated by or in co-operation with the ward or team management. The management must also ensure that nurses are given sufficient time off of routine work as is common in medical devices integration. Similarly, time off for training sessions is essential to ensure safe handling and becoming key user for the device. Manufacturers should provide comprehensive support and assistance during the integration process. Repeated training over time should also be offered in order to train more or newly hired nurses in addition to the core team.

## Conclusion

The healthcare systems face multiple challenges like the shortage of skilled workers. If implemented well and sustainably, robotic systems offer the opportunity to provide higher frequency training for patients while relieving nurses of the physical effort. For accomplishing this, the prerequisites and the conditions of the respective hospital/ ward must be checked and adapted prior to the purchase. When integrating the device, it is recommended to involve the concerned end-users fully and provide sufficient time off to train the handling of the device. Support from the manufacturers is essential. For the future, consideration should be given to how the innovative therapy is included in standard care and funding.

## Data Availability

The datasets used and analysed during the current study are available from the corresponding author on reasonable request.

## References

[1] International Agency for Research on Cancer. Cancer Tomorrow. Estimated number of new cases from 2020 to 2040, both sexes, age [0–85+]. 2020. https://gco.iarc.fr/tomorrow/en/dataviz/bars?mode=population. Accessed 8 Aug 2022.

[2] Feigin VL, Stark BA, Johnson CO, et al. Global, regional, and national burden of stroke and its risk factors, 1990–2019: a systematic analysis for the global burden of Disease Study 2019. Lancet Neurol. 2021;795–820. 10.1016/S1474-4422(21)00252-0. 10.1016/S1474-4422(21)00252-0 PMC8443449 34487721

[3] Malekahmadi M, Moradi Moghaddam O, Islam SMS, Tanha K, Nematy M, Pahlavani N, Firouzi S, Zali MR, Norouzy A. Evaluation of the effects of pycnogenol (French maritime pine bark extract) supplementation on inflammatory biomarkers and nutritional and clinical status in traumatic brain injury patients in an intensive care unit: A randomized clinical trial protocol 2020. doi:10.1186/s13063-019-4008-x. 10.1186/s13063-019-4008-x PMC7014642 32046747

[4] Diserens K, Moreira T, Hirt L, Faouzi M, Grujic J, Bieler G (2012). Early mobilization out of bed after ischaemic stroke reduces severe complications but not cerebral blood flow: a randomized controlled pilot trial. Clin Rehabil.

[5] Chiang LL, Wang LY, Wu CP, Wu HD, Wu YT (2006). Effects of physical training on functional status in patients with prolonged mechanical ventilation. Phys Ther.

[6] Bernhardt J, Dewey H, Collier J, Thrift A, Lindley R, Moodie M, Donnan G (2006). A very early Rehabilitation Trial (AVERT). Int J Stroke.

[7] Beyer J, Seidel EJ (2017). Frührehabilitation ist erstes Glied einer nahtlosen Rehabilitationskette. [Acute Care Rehabilitation is the First Link in a chain of Rehabilitation Interventions]. Rehabilitation (Stuttg).

[8] Bein T, Bischoff M, Brückner U, Gebhardt K, Henzler D, Hermes C (2015). Kurzversion S2e-Leitlinie - “Lagerungstherapie und Frühmobilisation zur Prophylaxe oder Therapie von pulmonalen Funktionsstörungen”. [Short version S2e guidelines: “Positioning therapy and early mobilization for prophylaxis or therapy of pulmonary function disorders”]. Anaesthesist.

[9] Hermans G, Van den Berghe G. Clinical review: intensive care unit acquired weakness.Crit Care. 2015. 10.1186/s13054-015-0993-7 PMC4526175 26242743

[10] Miranda Rocha AR, Martinez BP, Maldaner da Silva VZ, Forgiarini Junior LA. Early mobilization: Why, what for and how?Med Intensiva. 2017. 10.1016/j.medin.2016.10.003 28283324

[11] Berney S, Elliot D, Denehy L. ICU-acquired weakness - a call to arms (and legs).Crit Care Resusc. 2011. 21355821

[12] Herridge MS, Tansey CM, Matté A, Tomlinson G, Diaz-Granados N, Cooper A (2011). Functional disability 5 years after acute respiratory distress syndrome. N Engl J Med.

[13] Hermans G, de Jonghe B, Bruyninckx F, van den Berghe G. Interventions for preventing critical illness polyneuropathy and critical illness myopathy. Cochrane Database Syst Rev. 2009;CD006832. 10.1002/14651858.CD006832.pub2. 10.1002/14651858.CD006832.pub2 19160304

[14] Morris PE, Goad A, Thompson C, Taylor K, Harry B, Passmore L (2008). Early intensive care unit mobility therapy in the treatment of acute respiratory failure. Crit Care Med.

[15] Winkelman C, Peereboom K. Staff-perceived barriers and facilitators.Crit. Care Nurse. 2010:2. 10.4037/ccn2010393 20360441

[16] McWilliams DJ, Pantelides KP. Does physiotherapy led early mobilisation affect length of stay in ICU?ACPRC Journal. 2008:5–10.

[17] Barber EA, Everard T, Holland AE, Tipping C, Bradley SJ, Hodgson CL. Barriers and facilitators to early mobilisation in Intensive Care: a qualitative study. Aust Crit Care. 2015;28. 10.1016/j.aucc.2014.11.001. 177 – 82; quiz 183. 10.1016/j.aucc.2014.11.001 25533868

[18] Calabrò RS, Naro A, Russo M, Leo A, Balletta T, Saccá I (2015). Do post-stroke patients benefit from robotic verticalization? A pilot-study focusing on a Novel Neurophysiological Approach. Restor Neurol Neurosci.

[19] Villafañe JH, Taveggia G, Galeri S, Bissolotti L, Mullè C, Imperio G, Valdes K, Borboni A, Negrini S. Efficacy of Short-Term Robot-Assisted Rehabilitation in Patients With Hand Paralysis After Stroke: A Randomized Clinical Trial. Hand (N Y). 2018. 10.1177/1558944717692096 PMC5755871 28719996

[20] Taveggia G, Borboni A, Salvi L, Mulé C, Fogliaresi S, Villafañe JH, Casale R. Efficacy of robot-assisted rehabilitation for the functional recovery of the upper limb in post-stroke patients: a randomized controlled study.Eur J Phys Rehabil Med. 2016. 27406879

[21] Huebner L, Schroederh I, Kraft E, Gutmann M, Biebl J, Klamt A, Frey J, Warmbein A, Rathgeber I, Eberl I, Fischer U, Scharf C, Schaller S, Zoller M. Frühmobilisation auf der Intensivstation – Sind robotergestützte Systeme die Zukunft? Anaesthesiologie 2022. doi:10.1007/s00101-022-01130-x. 10.1007/s00101-022-01130-x 35925160

[22] Just IA, Fries D, Loewe S, Falk V, Cesarovic N, Edelmann F, Feuerstein A, Haufe FL, Xiloyannis M, Riener R, Schoenrath F (2022). Movement therapy in advanced heart failure assisted by a lightweight wearable robot: a feasibility pilot study. ESC Heart Fail.

[23] Klamt AC, Schmidbauer L, Warmbein A, Rathgeber I, Fischer U, Eberl I (2021). Very early Robot-Assisted mobilization of Intensive Care Patients - A Scoping Review. Stud Health Technol Inform.

[24] Bertelsen AS, Storm A, Minet L, Ryg J. Use of robot technology in passive mobilization of acute hospitalized geriatric medicine patients: a pilot test and feasibility study.Pilot Feasibility Stud. 2020. 10.1186/s40814-019-0545-z PMC6943926 31921434

[25] Shaw J, Agarwal P, Desveaux L, Palma DC, Stamenova V, Jamieson T (2018). Beyond “implementation”: digital health innovation and service design. NPJ Digit Med.

[26] Greenhalgh T, Wherton J, Papoutsi C, Lynch J, Hughes G, A’Court C (2017). Beyond adoption: a New Framework for Theorizing and evaluating nonadoption, abandonment, and Challenges to the Scale-Up, Spread, and sustainability of Health and Care Technologies. J Med Internet Res.

[27] Zerth J, Jaensch P, Müller S, Technik.Pflegeinnovation und Implementierungsbedingungen:157–72. doi:10.1007/978-3-662-63107-2_11.

[28] Fernandez ME, Hoor GA ten, van Lieshout S, Rodriguez SA, Beidas RS, Parcel G et al. Implementation Mapping: Using Intervention Mapping to Develop Implementation Strategies. Front Public Health. 2019;7:158. doi:10.3389/fpubh.2019.00158. 10.3389/fpubh.2019.00158 PMC6592155 31275915

[29] Craig P, Dieppe P, Macintyre S, Michie S, Nazareth I, Petticrew M. Developing and evaluating complex interventions:: Following considerable development in the field since 2006, MRC and NIHR have jointly commissioned an update of this guidance to be published in 2019. 2019. https://www.mrc.ac.uk/documents/pdf/complex-interventions-guidance/.

[30] Tong A, Sainsbury P, Craig J. Consolidated criteria for reporting qualitative research (COREQ): a 32-item checklist for interviews and focus groups.International J Qual Health Care. 2007. 10.1093/intqhc/mzm042 17872937

[31] Bogner A, Littig B, Menz W. Das Experteninterview. Theorie, Methode, Anwendung; 2005.

[32] DePoy E, Gitlin LN (2016). Introduction to research: understanding and applying multiple strategies.

[33] VERBI – Software. Consult. Sozialforschung. GmbH. MAXQDA 2022. www.maxqda.de.

[34] Bengtsson M (2016). How to plan and perform a qualitative study using content analysis. NursingPlus Open.

[35] Creswell JW, Plano Clark VL (2018). Designing and conducting mixed methods research.

[36] Kvale S (1996). Interviews: an introduction to qualitative research interviewing.

[37] Flick U, Baur N, Blasius J (2019). Gütekriterien qualitativer Sozialforschung. Handbuch Methoden der empirischen Sozialforschung: Band 1.

[38] Flick U, von Kardorff E, Steinke I (2009). Qualitative Forschung: Ein Handbuch.

[39] Speziale HS, Streubert HJ, Carpenter DR. Qualitative research in nursing: advancing the humanistic imperative. 5th ed. Lippincott Williams & Wilkins; 2011.

[40] Polit DF, Beck CT (2016). Nursing research: generating and assessing evidence for nursing practice.

[41] Birken SA, Powell BJ, Presseau J, Kirk MA, Lorencatto F, Gould NJ (2017). Combined use of the Consolidated Framework for implementation research (CFIR) and the theoretical domains Framework (TDF): a systematic review. Implement Sci.

[42] Hübner U (2010). Pflegeinformatik: Mehrwert für die Patientenversorgung. Dtsch Ärzteblatt.

[43] Daumann S (2018). Wundmanagement und Wunddokumentation.

[44] Vermeersch P, Sampsel DD, Kleman C. Acceptability and usability of a telepresence robot for geriatric primary care: A pilot. 2018;Geriatric nursing. 10.1016/j.gerinurse.2015.04.009 25959035

[45] Kerssens C, Kumar R, Adams AE, Knott CC, Matalenas L, Sanford JA, Rogers WA, Kerssens C, Kumar R, Adams AE, Knott CC, Matalenas L, Sanford JA, Rogers WA. Personalized technology to. Personalized technology to support older adults with and without cognitive impairment living at home. American journal of Alzheimer’s disease and other dementias 2015. doi:10.1177/1533317514568338. 10.1177/1533317514568338 PMC4819239 25614507

[46] Servaty R, Möhler R, Kersten A, Brukamp K, Müller M. Barriers and facilitators of implementing robotic systems in nursing care. In: Pflegeinnovationszentrum, editor. Clusterkonferenz 2018; 2018.

[47] Waibel AK, Holl F, Swoboda W, Fotteler M. Chances and Risks of Using Robotic Assistance Systems in Early Neurological Rehabilitation: A Qualitative Analysis. Studies in Health Technology and Informatics. 2022. 10.3233/SHTI220758 35773904

[48] Grunow JJ, Nydahl P, Schaller SJ (2022). Mobilisation auf Intensivstationen: Intensivpflegezimmer und Medizintechnik können helfen. [Mobilization of Intensive Care Unit Patients: how can the ICU rooms and Modern Medical Equipment help?]. Anaesthesiol Intensivmed Notfallmed Schmerzther.

[49] Warmbein A, Schroeder I, Mehler-Klamt A, Rathgeber I, Huber J, Scharf C (2022). Robot-assisted early mobilization of intensive care patients: a feasibility study protocol. Pilot and Feasibility Studies.

